# The effects of COVID-19 on reproductive, maternal, neonatal, and child health service provision and utilisation: qualitative results from Burkina Faso and Mozambique

**DOI:** 10.7189/jogh.16.04036

**Published:** 2026-03-06

**Authors:** Kelsey Zack, Souleymane Zorome, Midália Uamba, Kadidiatou Kadio, Sérgio Mahumane, Moussa Bougma, Milton Sengo, Hannah Fritz, Celso Monjane, Abdoulaye Maïga, Almamy Malick Kante, Ivalda Macicame, Agbessi Amouzou

**Affiliations:** 1 Johns Hopkins University Bloomberg School of Public Health, Baltimore, Maryland, USA; 2 Institut Supérieur des Sciences de la Population, Ouagadougou, Kadiogo, Burkina Faso; 3 Institut de Recherche en Sciences de la Santé, Ouagadougou, Kadiogo, Burkina Faso; 4 Instituto Nacional de Saúde, Marracuene, Maputo, Mozambique

## Abstract

**Background:**

The COVID-19 pandemic disrupted country health systems and necessitated urgent actions to offset its effects on service provision, especially for vulnerable populations such as mothers and children. We aimed to analyse the experiences of healthcare workers in Burkina Faso and Mozambique, and the perceived effects of COVID-19 on reproductive, maternal, newborn, and child health (RMNCH) service provision and utilisation.

**Methods:**

We conducted key informant interviews with healthcare workers involved in direct patient care and managerial positions in two provinces in Burkina Faso (Kadiogo and Boulkiemdé) (n = 33) and three provinces in Mozambique (Maputo City, Maputo Province, and Nampula) (n = 66). We audio-recorded, transcribed, and coded the interviews using a deductive and inductive coding approach. We analysed perceptions of RMNCH service disruptions and compared the results between the two countries. We used an inductive analysis method.

**Results:**

The health systems in Burkina Faso and Mozambique reacted quickly and in a similar way to contain the COVID-19 pandemic. However, the adoption of COVID-19 activities and the implementation of rotational staff schedules may have slowed down the provision of services. Some services, such as antenatal care and child nutritional services, were limited. In both countries, respondents reported that unforeseen patient costs, such as for face masks, shortages in child medications, and fear from patients of getting COVID-19 virus at health facilities appeared to have hindered service utilisation.

**Conclusions:**

The COVID-19 pandemic did not appear to have ceased the availability of or cause substantial disruptions to RMNCH services at health facilities in either country, but our findings showed that key informants perceived the pandemic did influence the reorganisation of health services, and the provision and utilisation of RMNCH services.

As of May 2023, COVID-19 is no longer considered a public health emergency of international concern by the World Health Organization (WHO), after first emerging in late 2019 [1–3]. In low- and middle-income countries, where health systems are generally weaker, with less access to medical resources and care, and with higher rates of malnutrition and infectious diseases, the COVID-19 pandemic was predicted to have a particularly detrimental impact [4]. With the WHO declaring COVID-19 a public health emergency of international concern in January 2020 and subsequently a global pandemic in March 2020, governments began adopting and developing guidelines and deploying public health responses to mitigate the pandemic's effects at both national and local levels [5–9].

In sub-Saharan Africa, many countries implemented a variety of measures to combat the spread of COVID-19: entry and border restrictions, social distancing measures, mandatory use of face masks in public spaces, prohibition of large-scale events, and school closures [10–12]. Additionally, rapid plans and guidelines proposed by governing bodies in response to the pandemic were enacted in hospitals, health centres, and communities.

Across the region, there were concerns about how a global pandemic could affect routine health services, especially for mothers and children [13,14]. Lessons from past outbreaks suggest that changes and disruptions to health services, community health activities, medical supply chains, and shifts in social perceptions can have lasting consequences on populations, such as decreased rates of care seeking and healthcare utilisation [9,14–16]. Qualitative studies on past disease outbreaks have also reported that factors such as fear amongst a population of contracting a disease can decrease rates of health service utilisation [7,8,16].

In 2020, Burkina Faso had a reported total population of 21.5 million, with women comprising 50.2% of the population, and Mozambique had a reported total population of 31.2 million, with women comprising 50.9% of the population [17–22]. Both countries had high fertility rates in 2020: 4.9 births per woman in Burkina Faso, and 4.7 births per woman in Mozambique [23,24]. We report on the experiences of health workers in direct patient care roles and managerial positions, and their perceptions of how the COVID-19 pandemic affected reproductive, maternal, newborn, and child health (RMNCH) service provision and utilisation in Burkina Faso and Mozambique.

## METHODS

### Study setting and design

In this qualitative study, we focused on key informants’ perceptions of the impact of COVID-19 on RMNCH services in Burkina Faso and Mozambique. The countries were chosen for two reasons: strong in-country partnerships between Johns Hopkins University and the existence of on-going collaborative activities, including the implementation of a sample mortality surveillance system, the Countrywide Mortality Surveillance for Action system, in Mozambique, and the Real Accountability: Data and Analysis for Results project in Burkina Faso, that implemented a large household survey from February to March 2020, prior to the COVID-19 pandemic.

We implemented the study in two provinces in Burkina Faso, Kadiogo and Boulkiemdé, and in three provinces in Mozambique, Maputo City, Maputo Province, and Nampula. Kadiogo Province in Burkina Faso is predominantly urban in the Central administrative region, and Boulkiemdé Province is mainly rural and located in the Western-Central administrative region [25]. As reported in 2019, Kadiogo Province has a population of about three million, and Boulkiemdé Province has a population of about 690 000 [25]. Maputo City and Maputo Province are in the Southern region of Mozambique, while Nampula Province is in the Northern region. As reported in the 2017 national census, Maputo City, the capital of the country, has a total population of about 1.1 million people, Maputo Province has a total population of about 2.3 million, and Nampula Province, the most populated province in Mozambique, has a total population of about 6.3 million [26].

### Selection and description of participants

We interviewed 33 key informants in Burkina Faso and 66 in Mozambique. Key informants consisted of health workers and health managers. Health workers included professionals providing direct patient care at the facility level, such as nurses, midwives, and health officers. Health managers were individuals who held managerial roles at the provincial or national levels, such as hospital managers, health unit directors, and government officials. We selected key informants through purposive sampling: only individuals who were actively working as health workers, or as health managers in the selected provinces, or at the national level were asked to participate in our study. We addressed potential selection bias by engaging with key informants across a range of service areas and departments, including midwifery, general nursing, maternal and child health, nutrition, as well as managerial, administrative, and logistical roles. In Burkina Faso, we interviewed 15 health workers and 18 health managers. In Mozambique, we interviewed 57 health workers and 9 health managers. To maintain anonymity in our results, we labelled key informant roles as Health Worker or Health Manager.

### Data collection and measurements

We conducted in-depth, semi-structured individual interviews in both countries. Both countries used the same interview guide for data collection, with adaptations to country-specific contexts and translations into French in Burkina Faso and Portuguese in Mozambique. This guide addressed themes related to respondents' perceptions of the effects of the pandemic on the availability and use of health services, perceptions of the health system's level of preparedness for the pandemic, the health system's plans and responses, attitudes and perceptions towards COVID-19 measures, and the challenges faced in maintaining health services in the context of a global pandemic. We recorded all interviews in both countries using voice recorders and conducted note-taking and non-participatory observations of practices in the health facilities.

In each country, we selected and trained four interviewers with experience in qualitative data collection to carry out data collection. In Burkina Faso, the training lasted three days, and in Mozambique, seven days. We collected the data from 11 May to 13 June 2022 in Burkina Faso, and from 21 November 2022 to 27 January 2023 in Mozambique. In Mozambique, we conducted four additional interviews at the Ministry of Health and Maputo Central Hospital, the largest hospital in the country, in Maputo City from 17 to 23 July 2023.

The Burkina Faso and Mozambique study teams worked separately, using their own data and following similar analysis approaches. Audio recordings of each interview were transcribed in the respective language of the interview, French in Burkina Faso and Portuguese in Mozambique. We compared transcribed interviews with audio recordings to ensure accuracy and data quality. We used an integral model for word-by-word transcription. We then transferred the data to a password-protected computer for storage. We imported transcripts into NVivo, version 20 (Lumivero, Denver, Colorado, USA) for analysis. Both country teams began with the same non-rigid coding guide, which was developed based on the study objectives and themes outlined in the interview guides. The non-rigid coding guide allowed for flexibility across the two countries and their varying contexts, and for emerging codes and sub-topics to be added iteratively. We used a mixed approach of deductive and inductive coding. The qualitative research teams carried out and discussed open coding to clarify certain codes. We used interview transcriptions and notes taken during interviews during the analysis process.

The data analysis approach for both countries consisted of identifying text extracts, organising them according to the coding frame and study objectives, systematically exploring the data, grouping texts into themes, grouping themes into categories, and a final review of the theme groupings and code categories. We adopted an inductive analysis method to examine and synthesise the data.

We categorised the interview data further by themes that included perceived effects on either service provision or utilisation. Themes pertaining to service provision included health staff adoption of COVID-19 activities, the reorganisation of health services, and budget reallocation. Themes pertaining to service utilisation included financial burdens on facility patients and fear of virus transmission.

## RESULTS

### Effects of COVID-19 on RMNCH service provision

#### Maintaining routine service provision

In both countries, key informants reported changes to the provision of routine health services during the pandemic to prevent the spread of COVID-19, noting that some RMNCH consultation periods were extended and some services slowed. Health workers interviewed in both countries reported having to take on additional COVID-19-related tasks. Despite the increased intensity of activities during the COVID-19 period, the respondents reported that they continued to provide the same daily health services.

Yes, we provided clinical care to patients. We didn't stop giving birth, we didn't stop curative consultations; we continued what we should be doing. – *(Health Worker 1, Burkina Faso)*

(COVID-19) affected the flow and demand for services, but the services were (still) here. – *(Health Manager 1, Mozambique)*

In Burkina Faso, health personnel in all sectors received sanitary equipment to combat COVID-19, as well as additional financial support for the medical teams involved in prevention, control, testing, and vaccination activities.

Yes, there was support. We’ve been provided with materials, and even goodwill donors have given us protective equipment. The Government also donated bleach, soap, gloves, masks and bibs. – *(Health Worker 2, Burkina Faso)*

In both Burkina Faso and Mozambique, most key informants in managerial roles reported participating in training on handwashing techniques, the use of masks and personal protective equipment (PPE), and how to provide care to specific groups under prevention measures, such as pregnant women and patients with chronic diseases. However, there were some reports in both countries that health workers in direct patient care received limited or no formal training on COVID-19 prevention and procedures.

Not all the staff went, maybe only our bosses went there, and then they shared the information with us. – *(Health Worker 1, Mozambique)*

There was not a real training. There were informational posters, and we were told to be careful because health personnel were dying. – *(Health Worker 2, Mozambique)*

#### Reorganisation of health services

Health services in both countries were reorganised to follow infection prevention measures, which were implemented for both healthcare staff and patients. Measures included mask use, social distancing, and handwashing. Health facilities reorganised staff schedules and extended consultation periods for some RMNCH services, such as antenatal care (ANC), family planning, and child weighing, to prevent exposure to the virus and to focus efforts on COVID-19 cases. In both Burkina Faso and Mozambique, health staff interviewed shared that a rotational system was implemented by dividing staff into working groups. The goal of the rotational system was to prevent potential widespread infection amongst health staff and to ensure that health staff continued to provide routine services.

We set up teams so that not all staff were there at the same time, exposed to the same risks. We’ve made sure that there are a few rotations so that people won’t run into each other. – *(Health Worker 3, Burkina Faso)*

We made a rotational schedule in the COVID sector. We made a rotational schedule, and I was also working in the aid bank. – *(Health Worker 2, Mozambique)*

Health services were also reorganised in accordance with the COVID-19 prevention measures, which led to additional stress on health staff and reported service delays. Although fewer health staff were working at any given time due to rotational schedules, the provision of RMNCH services did not stop entirely, but wait times increased.

As the number of staff is reduced because of the reorganisation, it can really cause inconvenience for the women in the form of long waits. – *(Health Worker 4, Burkina Faso)*

(…) we started to adopt an appointment system to avoid over-crowding of the patients themselves. – *(Health Worker 3, Mozambique)*

Consultations were no longer monthly; they became quarterly for children as well as pregnant women. – *(Health Worker 4, Mozambique)*

The implementation and application of prevention measures had slowed and overcrowded health services due to the rearrangement of health staff, ultimately reducing the provision of some RMNCH services, notably sexual and reproductive health services. Decreased service utilisation was also reported in both countries due to the rearrangement of health services and the lengthening of appointment times, but most RMNCH services were ongoing, with minimal disruptions due to COVID-19.

#### Budget reallocation

Key informants in both countries noted that maternal and child health programmes and services were prioritised within the health system, with funding for such services remaining consistent through government measures and/or external funding from government partnerships with non-government organisations. In Burkina Faso, funds for RMNCH services in health units are channelled through the Ministry of Health and are used directly to cover expenses, ensuring these services are free of charge for patients. This consistent funding structure ensured that RMNCH services remained free of charge even during peak COVID-19 periods. It is unclear whether the budget for RMNCH services was reallocated during our study.

Maternal and child health is one of our country's health priorities, and the focus in terms of funding was good - funding was more oriented towards maternal and child health. But with the advent of COVID-19, (other) funds were redirected to the fight against COVID-19. – *(Health Manager 1, Burkina Faso)*

In Mozambique, many maternal and child health programmes operate with substantial support from other partners. Thus, it was reported that the pandemic did not directly affect funding for RMNCH programmes. It was also reported that funds were allocated for COVID-19 prevention activities in health units, but it is unclear whether these were existing funds within the health system that were reallocated or whether new funding was received. Health staff mentioned that funds allocated to carry out these COVID-19 activities were insufficient in the long term because of the need to replenish PPE stock.

We had funds to carry out (COVID) activities, but during the COVID-19 peak we had to take these funds to buy masks and gloves for health workers to use. – *(Health Manager 2, Mozambique)*

While funding for RMNCH programmes was reported to have remained consistent in both countries, changes in the logistics and operations of RMNCH services caused by COVID-19 restrictions affected service provision, including reductions in the frequency of ANC and family planning consultations and in child nutrition services.

### Perceived effects of COVID-19 on RMNCH service utilisation

#### Financial burdens

During the pandemic, some supplies and medicines that would normally be covered by health facilities fell to the patient's responsibility. It is unclear whether face masks were offered to patients free of charge in either country, and the requirement to pay for face masks in some cases is considered to have created a financial barrier for some patients in accessing RMNCH services during the pandemic. Interviewees in Burkina Faso reported that patients who could not afford a face mask to enter health facilities often left without receiving the intended health service.

Sometimes they come without their masks, so we make them aware of the problem and ask them to pay for their masks. Others say they don’t have the money to pay for the masks, so they leave. – *(Health Worker 5, Burkina Faso)*

The requirement to wear masks to access health services, when health facilities could not provide them, is perceived to have acted as a financial barrier to accessing RMNCH services for some patients. As a result, many women likely chose not to seek RMNCH services during the pandemic if they did not have access to, or could not afford, face masks. In Mozambique, it was also reported that some supplements and medicines were unavailable due to supply-chain issues in production, and patients were advised to seek medicines elsewhere, such as at privately owned pharmacies.

A lot of essential medicines for newborns were restricted, and there were a lot of shortages that we had to adapt to. We had to ask the families to buy it themselves. – *(Health Manager 3, Mozambique)*

Logistical difficulties in procuring medicines began at the central level of the health system but had more noticeable effects on health facilities. As a result, health facilities faced shortages of essential medicines for newborns and children, and the responsibility for obtaining and purchasing such medicines elsewhere fell to caregivers. Overall, in both countries, rising costs of items such as face masks and certain medicines were reported to have influenced patient utilisation of RMNCH services.

#### Fear

Key informants reported being afraid themselves of falling sick with COVID-19 and reported that patients perceived health facilities as a place where they could ‘catch’ COVID-19, creating a fear amongst communities to visit hospitals and health centres.

The fact that we are health professionals does not take away our fear, we were afraid. – *(Health Worker 5, Mozambique)*

Key informants in Mozambique reported experiencing fear while working at health facilities due to limited information on COVID-19 at the beginning of the pandemic and the additional stress that COVID-19 brought to their daily lives. Key informants reported experiencing fear from seeing colleagues contract the virus and falling ill, and reported feeling apprehensive about working in an environment where they may be more exposed to COVID-19. Key informants in Burkina Faso reported similar experiences.

There was a change, yes, the number of consultations reduced a lot. People were afraid to leave their homes to go to the health units. – *(Health Worker 6, Mozambique)*

In Mozambique, key informants reported a slight change in the utilisation of some RMNCH services, namely in facility-based deliveries, consultations for contraceptive services, and child nutrition monitoring services. This change in demand and in attendance was attributed to patients’ fear of contracting the virus at health facilities. Similar patterns were reported in Burkina Faso, where patients also perceived health centres as places where they could contract COVID-19, leading them to avoid visiting health facilities altogether during the pandemic. Key informants in Burkina Faso also reported that this sense of fear led to a reduction in the number of patients requesting consultations for RMNCH services.

## DISCUSSION

We highlighted key informants’ reports of the effects of COVID-19 on RMNCH service provision and utilisation in health facilities in Burkina Faso and Mozambiqu. Key informants included health workers in direct patient care and healthcare managers. All key informants were involved in providing RMNCH services in their respective roles.

We also highlighted changes implemented in health facilities during COVID-19 that affected RMNCH service provision: the addition of COVID-19 tasks and activities, and the implementation of rotational health staff schedules. The addition of COVID-19 tasks led to the re-arrangement of health staff in both countries to prevent infection and maintain the health staff workforce. This re-arrangement is common, as reported in other studies during past epidemics and pandemics [3,27]. In a study conducted in Ethiopia, looking at the effects of COVID-19 on essential health services, it was found that health workers were often reassigned to focus their efforts on supporting COVID-19 services and that a leading cause of staff shortages in the study area was due to high rates of healthcare worker mortality and morbidity [28]. Despite the requisition of some health workers for COVID-19-specific activities in Burkina Faso and Mozambique, we found that most RMNCH services remained operational throughout the pandemic, although we observed changes in the frequency of some services, such as ANC consultations and child nutrition services.

According to health managers interviewed, budgets for RMNCH programmes in both countries were, by and large, not affected by the advent of COVID-19. With a generally weaker health system infrastructure and a high prevalence of other diseases, such as HIV, it was predicted that many countries in sub-Saharan Africa would endure devastating outcomes from COVID-19, which may have necessitated urgent changes in national health budget allocations and funding to address [29]. However, despite these predictions, these changes did not generally occur as expected. While there were no substantial reports of budget reallocation for RMNCH services and programmes in either country, there were reports of insufficient funding for PPE in Mozambique. The consistent funding of RMNCH programming during peak pandemic periods ultimately highlights the continued prioritisation of RMNCH programmes and services in Burkina Faso and Mozambique.

We also revealed that perceived fears of getting sick were prevalent among both health staff and patients, and that these fears affected the utilisation of RMNCH services in study areas in Burkina Faso and Mozambique. Shared experiences from past outbreaks, such as the 2014 Ebola outbreak in West Africa and the 2003 global severe acute respiratory syndrome epidemic, show that fear of infection amongst patients and communities is a common occurrence that affects health service utilisation. In other studies, decreases in healthcare utilisation and reluctance to visit health facilities were linked to lack of knowledge of the source of disease and fears of contracting viruses at health facilities [28,30,31]. In a 2022 study conducted in Nampula, Mozambique, authors reported that early ANC visits increased post-pandemic, and that these results may be due to health staff in the study area effectively communicating and encouraging the use of available services during lockdown periods [32]. These communication efforts may also have been supported by effective health staff training on COVID-19 and health facility procedures; however, according to some key informants, such training was lacking in both countries. Given the consistent theme of fear across past research and the current study, this finding calls for health systems to financially and programmatically prioritise training and information-sharing interventions for all health workers for future outbreaks and pandemics.

According to our results, inferred financial burdens found at health facilities during the pandemic were another factor that had perceived effects on RMNCH health service utilisation. We found that the mandatory use of face masks, which often required patients to purchase them themselves to comply with infection prevention measures, may have affected patients’ willingness to seek RMNCH services. Other instances in which essential medicines were not available at government-funded health facilities, which may have led patients to seek them elsewhere, may also have affected RMNCH service utilisation. We know from other studies that COVID-19-related lockdowns caused a loss of income for some people and ultimately prevented them from paying for some health services [28,30,31]. Overall, financial stressors caused by the pandemic, whether related to buying face masks, medications, or other factors not explored in the present study, were perceived to have affected RMNCH service utilisation in the study areas.

Our study has some limitations. We based our study on reported effects of the COVID-19 pandemic on RMNCH services as perceived by a purposively selected sample of key informants. We have tried to select a diverse sample and collected data until saturation. However, being health workers and managers, key informants’ self-reports may be tainted by their own behaviour and experiences. The consistency between our findings and those of other similar studies reinforces the accuracy of the reporting. In addition, our quantitative analysis of the impact of COVID-19 on coverage of RMNCH interventions published elsewhere shows no significant effects [33].

## CONCLUSIONS

Despite previous predictions on how the COVID-19 pandemic was expected to affect countries in sub-Saharan Africa, the present study concluded that COVID-19 ultimately did not cease the availability of or cause significant disruptions to RMNCH services in Burkina Faso and Mozambique. Although we did not find significant cessations and disruptions to RMNCH services in either country, key informants’ perspectives are insightful into the perceived effects of COVID-19 on RMNCH service provision and utilisation in both countries. We recognise that there may be additional cultural, economic, and systemic factors in both countries that may have shaped key informant perceptions of behaviour in service provision and utilisation during the pandemic, as reported in this study. Further research is required to explore how other socioeconomic factors in the study areas may have affected RMNCH service provision and utilisation from both health worker and patient perspectives. While the conclusions from this study may reflect findings from past epidemics and pandemics, it provides insights into how the most recent global pandemic affected two select countries in sub-Saharan Africa, a region that was originally predicted to be severely affected by COVID-19 compared to other regions globally. Findings from this study can be used to support building health systems that are resilient, ready, and organised to handle future pandemics.
